# Editorial Comment: Does successful urethral calibration rule out significant female urethral stenosis? confronting the confounder- an outcome analysis of successfully treated female urethral strictures

**DOI:** 10.1590/S1677-5538.IBJU.2020.0857.1

**Published:** 2021-07-20

**Authors:** André G. Cavalcanti

**Affiliations:** 1 Universidade Federal do Estado do Rio de Janeiro Departamento de Cirurgia Geral e Especializada Rio de JaneiroRJ Brasil Departamento de Cirurgia Geral e Especializada, Universidade Federal do Estado do Rio de Janeiro - UNIRIO, Rio de Janeiro, RJ, Brasil

## COMMENT

Female urethral stenosis is a condition that is probably underdiagnosed and many women spend years with their symptoms before receiving appropriate treatment. In addition, many women with a suspected urethral obstruction are placed in chronic dilation without adequate and definitive treatment. Different techniques of urethroplasty have already been described using flaps or grafts (vaginal or buccal) in different positions (ventral or dorsal). All these techniques have been showing very acceptable results of success with low rates of urinary incontinence. Undoubtedly superior results when compared to dilation, which can often be considered the cause or the worsening factor of the stenosis.

In this very original study, the authors retrospectively evaluate the preoperative data of 16 patients who underwent urethroplasty. In 13/16 patients (1), the calibration of the urethra was successful in the preoperative period, and despite this, these patients presented obstruction in the evaluation with video-urodynamics and signs of urethral stenosis on ureteroscopy. This is an interesting data that demonstrates that only an adequate caliber does not rule out the presence of obstruction and that these patients show a significant improvement in the voiding pattern when submitted to urethroplasty even with a 14 Fr caliber dilation. It is a situation that sometimes occurs with these patients, where we often do not find a picture of dense fibrosis, due to the anatomical characteristics of the female urethra already described in the text.

Many patients will certainly be left in a non-surgical treatment with a high impact on quality of life despite the fact that dilation is possible, thus the study expands the need for an adequate and thorough investigation in patients with suspected bladder outlet obstruction and takes into account the advantages of the video-urodynamic study, which, although not necessary in all patients, is likely to be useful in a significant group.
